# Structural Responses of Nucleic Acids to Mars-Relevant Salts at Deep Subsurface Conditions

**DOI:** 10.3390/life12050677

**Published:** 2022-05-02

**Authors:** Jim-Marcel Knop, Sanjib K. Mukherjee, Stewart Gault, Charles S. Cockell, Roland Winter

**Affiliations:** 1 Physical Chemistry I—Biophysical Chemistry, Faculty of Chemistry and Chemical Biology, TU Dortmund University, Otto-Hahn Street 4a, 44227 Dortmund, Germany; jim-marcel.knop@tu-dortmund.de (J.-M.K.); sanjib.mukherjee@tu-dortmund.de (S.K.M.); 2 UK Centre for Astrobiology, SUPA School of Physics and Astronomy, University of Edinburgh, James Clerk Maxwell Building, Peter Guthrie Tait Road, Edinburgh EH9 3FD, UK; gaults@tcd.ie (S.G.); ccockell@exseed.ed.ac.uk (C.S.C.)

**Keywords:** B-DNA, DNA-hairpin, perchlorates, brines, pressure, Mars, habitability

## Abstract

High pressure deep subsurface environments of Mars may harbor high concentrations of dissolved salts, such as perchlorates, yet we know little about how these salts influence the conditions for life, particularly in combination with high hydrostatic pressure. We investigated the effects of high magnesium perchlorate concentrations compared to sodium and magnesium chloride salts and high pressure on the conformational dynamics and stability of double-stranded B-DNA and, as a representative of a non-canonical DNA structure, a DNA-hairpin (HP), whose structure is known to be rather pressure-sensitive. To this end, fluorescence spectroscopies including single-molecule FRET methodology were applied. Our results show that the stability both of the B-DNA as well as the DNA-HP is largely preserved at high pressures and high salt concentrations, including the presence of chaotropic perchlorates. The perchlorate anion has a small destabilizing effect compared to chloride, however. These results show that high pressures at the kbar level and perchlorate anions can modify the stability of nucleic acids, but that they do not represent a barrier to the gross stability of such molecules in conditions associated with the deep subsurface of Mars.

## 1. Introduction

Investigating the chemical and physical limits of life and its associated biomacromolecules allows us to assess the habitability of extraterrestrial environments with respect to known life on Earth [1]. A large number of organisms thrive under extreme conditions on Earth, such as in the deep ocean, the subseafloor, in marine hydrothermal vents and volcanic environments, which are also suggested to be candidate locations for the origin of life. Extremophilic organisms on Earth, such as halophiles (salt-loving microorganisms), psychrophiles (cold-loving organisms) or piezophiles (pressure-loving microorganisms), as encountered in the deep sea where pressures up to about 1000 bar are encountered, are found in all three domains of life [1,2,3,4,5].

One of the requirements for life as we know it is the presence of liquid water. There is now abundant evidence of periods of surface water on ancient Mars, and more controversial suggestions of liquid water on present-day Mars, including in the deep subsurface [1,6,7,8]. In addition to any geochemical stressors, deep subsurface water on Mars would also be subjected to pressurization, which would be ∼1 kbar at the base of the cryosphere if it reached a depth of 10 km [6]. Such deep subsurface environments have been proposed to harbor high concentrations of dissolved salts, yet we know little about how such brines shape the conditions for life. One particular anion found ubiquitously on Mars is perchlorate, ClO_4_^−^ [9,10,11]. Perchlorate salts, such as Mg(ClO_4_)_2_, exhibit deep eutectic temperatures allowing for the presence of liquid water at temperatures as low as −70 °C. Perchlorates are known to act as chaotropes, perturbing the structure of water and its hydrogen bonding capacity [12], and might, hence, affect biomolecular hydration, structure, dynamics and function. To advance our understanding of the ability of subsurface environments of Mars to support life, we need to examine the combination of strong ionic effects imposed by these salts and high hydrostatic pressures (HHP).

Previous studies have demonstrated deleterious effects of perchlorates on the activity of enzymes such as α-chymotrypsin (α-CT) at ambient conditions [10,11,13]. Conversely, stabilizing salts, such as magnesium sulfate, MgSO_4_, have been shown to increase the activity and structural stability of α-CT [11]. We demonstrated that high pressures increase the enzymatic activity of α-CT, even in the presence of high perchlorate concentrations, and the results suggested that HHP may increase the habitability of environments under perchlorate stress. In another study, we investigated the binding of the small ligand 8-anilinonaphthalene-1-sulfonic acid (ANS) to the protein bovine serum albumin (BSA) at ambient and low temperatures, and at high pressure conditions in the presence of ions associated with the surface and subsurface of Mars [14]. We found that salts such as MgCl_2_ and MgSO_4_ only slightly affect protein–ligand complex formation. In contrast, Mg(ClO_4_)_2_ strongly affects the interaction between ANS and BSA, leading to a change in stoichiometry and strength of ligand binding. Remarkably, both a decrease in temperature and an increase in pressure favor the ligand binding process. Ligand binding studies of the complex formation between the ligand thioflavin T (ThT) and tRNA in the presence of Martian salts showed a strong reduction in the binding constant as well [15]. This effect is largely due to the interaction of ThT with the salt anions, which leads to a strong decrease in the activity of the ligand. Remarkably, the pressure favored ligand binding regardless of the type of salt [15]. Studies of the effect of Martian-like salts on biomolecular protein condensates based on liquid-liquid phase separation (LLPS) phenomena, which might also have played a significant role during protocell formation under prebiotic conditions, showed that the driving force for phase separation of dense protein solutions is not only sensitively dictated by the amino acid sequence of the polypeptide, but also strongly influenced by the type of salt and its concentration [16]. We showed that at high salinity, as encountered in Martian soil, short-range interactions, ion correlation (e.g., ion pairing) effects and hydrophobic interactions can sustain LLPS for suitable polypeptide sequences. We have also seen that spatial confinement, such as narrow pores in sediments or water pools, can dramatically stabilize the droplet phase [16]. In a subsequent study of these Martian salts on lipid-based compartments, viz. model biomembranes, we could show that the fluidity, lateral organization and morphology of lipid membranes are largely affected under extreme salt and pressure conditions relevant to Mars-like environments [17].

The effect of high concentrations of Mars-like salts on the stability of nucleic acids is still largely unknown. Pressure is known to have a small stabilizing, destabilizing or no effect on the structure of the B-DNA, depending on the temperature and salt concentration [18,19,20,21,22,23]. Recently, it was found that non-canonical nucleic acid structure, such as DNA-hairpins (DNA-HP) and G-quadruplexes, are much more pressure-sensitive, however [24,25,26,27,28,29,30,31,32]. Single-molecule Förster resonance energy transfer (sm-FRET) measurements [33] were used to directly measure the population distribution of DNA-HPs at HHP conditions. The pressure sensitivity of such structures is due to a conformational transition from a closed state to an open state, which is accompanied by a volume decrease, Δ*V*, in the order of −10 to −30 mL mol^−1^ [28,32].

Here, we present first data on the effect of high concentrations of Mg(ClO_4_)_2_ compared to NaCl and MgCl_2_ and high hydrostatic pressures on the conformational dynamics and stability of B-DNA and, as a representative of a non-canonical DNA structure, a DNA-HP. To this end, high-pressure UV-absorption spectroscopic and sm-FRET experiments were carried out.

## 2. Materials and Methods

The oligomeric nucleic acids used in this study were purchased from biomers.net (Ulm, Germany). The sequences are given below:

HP1: 5′-TGG CGA CGG CAG CGA GGC TTA GCG GCA (A)_30_ AGC CGC X-3′ (X is T-Atto 550); HP2: 5′-GCC TCG CYG CCG TCG CCA-3′ (Y is T-Atto 647N); A: 5′-GGA CTA GTC TAG GCG AAC GTT TAA GGC GAT CTC TGT TTA CAA CTC CGA-3′; B: 5′-TCG GAG TTG TAA ACA GAG ATC GCC TTA AAC GTT CGC CTA GAC TAG TCC-3′.

TrisHCl, TrisBase, NaCl, NaClO_4_, MgCl_2_ and Mg(ClO_4_)_2_ were purchased from Sigma-Aldrich (Darmstadt, Germany). The double-stranded DNA samples were prepared by mixing 20 µL of 100 µM solution of strand A and the same amount of the complementary strand B with 360 mM buffer containing 20 mM TrisHCl at pH 7.5 and 15 mM NaCl. The solution was heated to 95 °C for 5 min and subsequently cooled down to 25 °C at a rate of 1 °C min^−1^ to ensure proper annealing. For the UV-absorption measurements, 100 µL of this solution was added to 300 µL of buffer containing the required amount of salt to achieve a final concentration of 250 mM (or 15 mM) salt and 1.25 µM double-strand DNA.

Annealing of the DNA-HP for the single-molecule FRET microscopy experiments was carried out in a similar way. One microliter of each strand (HP1 and HP2) at a concentration of 100 µM was diluted in 100 µL of 20 mM TrisHCl pH 7.5 buffer with 15 mM NaCl. The annealed strands were kept at −80 °C for storage. Before the measurements, the 1 µM annealed stock solution was diluted to yield the desired low concentration for the single-molecule measurements of about 50–100 pM in 20 mM TrisHCl pH 7.5 buffer including the required concentration of salts.

The fluorescence microscopy setup and pressure apparatus used for the sm-FRET measurements was described in detail elsewhere [32]. The UV-spectroscopic measurements were performed using a Perkin Elmer Lambda 25 spectrometer. For the temperature dependent measurements at different pressure points, a home-made cylindrical stainless-steel pressure cell with two quartz glass windows (1 cm thickness) and water-thermostat for temperature control (Julabo Hl F32) was used. The path-length of the sample cell was 1 mm. Pressure was applied via a piston-pump using water as pressurizing medium. To separate the sample from the pressurizing medium, a drop of oil was applied to the connection of the pressure pump. Before the sample measurements, the spectrometer was blanked with the corresponding buffer.

## 3. Results and Discussion

**Figure 1:**
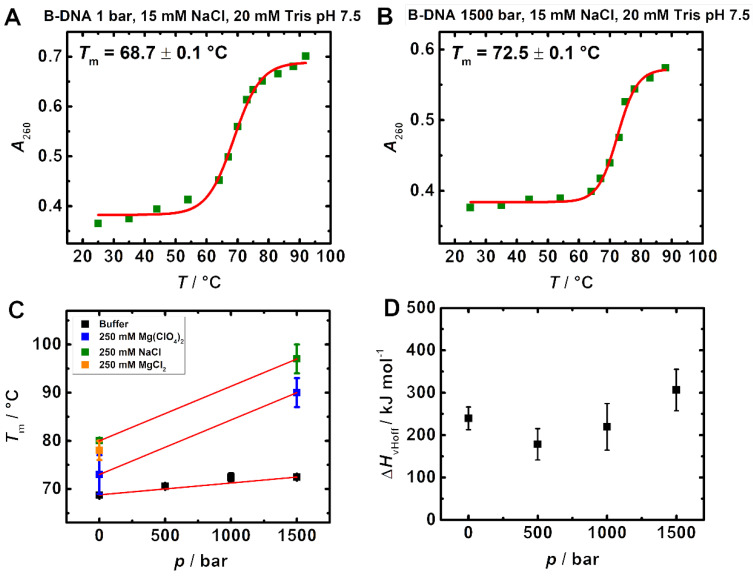
Pressure dependent melting curves of a 48 bp B-DNA. (**A**) shows the melting curve in buffer (15 mM NaCl, 20 mM Tris, pH 7.5) at 1 bar, (**B**) shows the melting curve at 1500 bar. (**C**) shows the pressure dependence of all *T*_m_-values obtained from Boltzmann fits including experiments carried out at high salt contents (250 mM NaCl, 250 mM MgCl_2_ and 250 mM Mg(ClO_4_)_2_). The maximum temperature measured was limited to 92 °C; therefore, *T*_m_ data at 1500 bar obtained from the Boltzmann fits can only be considered approximations, since a distinct second plateau was not reached. (**D**) shows the corresponding van’t Hoff melting enthalpies obtained for the measurement in buffer solution.

We show the combined effects of pressure and high concentrations of the salts MgCl_2_ and Mg(ClO_4_)_2_ on the stability, as expressed in increases in the melting temperature, *T*_m_, of two different classes of nucleic acid structures, a 48 bp B-DNA and an adenine DNA-HP. Figure 1A,B show, as representative examples, melting curves of the 48 bp B-DNA in buffer consisting of 15 mM NaCl, 20 mM Tris pH 7.5, at 1 bar and at 1500 bar, respectively. Figure 1C shows the pressure dependence of *T*_m_-values obtained including experiments carried out at high salt concentrations, viz. 250 mM NaCl, 250 mM MgCl_2_ and 250 mM Mg(ClO_4_)_2_. The addition of 250 mM NaCl increases *T*_m_ from 68.7 ± 0.1 °C to 80.0 ± 0.3 °C at ambient pressure. The salt MgCl_2_, at 250 mM, shows a similar strong stabilizing effect (*T*_m_ = 78 ± 2 °C). The addition of 250 mM Mg(ClO_4_)_2_ increases *T*_m_ from 68.7 ± 0.1 °C to 73 ± 4 °C only (Figure 1C), indicating a decreased stability compared to the presence of 250 mM NaCl.

The salts NaCl and Mg(ClO_4_)_2_ show a similar pressure dependence of the *T*_m_-values of the B-DNA (consisting of almost equal GC (23/48) and AT (25/48) bp) at 250 mM salt concentration. In both cases, *T*_m_ increases at a rate of about 10 °C/kbar. Conversely, in buffer solution we found the melting temperature to increase less dramatically with pressure (~3 °C/kbar) (Figure 1C). This finding is in good agreement with studies on poly(A-T) and poly(C-G) DNA, showing an increase in *T*_m_ of 3–4 °C/kbar [34].

The van’t Hoff melting enthalpy, Δ*H*_vHoff_, was obtained by using a Boltzmann fit of the melting curves for a two-state helix-to-coil transition. Figure 1D depicts the data for the measurement in buffer solution as a function of pressure, showing Δ*H*_vHoff_ values around 250 kJ mol^−1^ within the accuracy of the experiment (± 100 kJ mol^−1^). Owing to the absence of nice S-shaped melting curves at high salt concentration due to the high melting temperatures reached at high pressures, Δ*H*_vHoff_ values could not be determined for these samples. Using the Clapeyron equation

(1) $$\frac{dT_{m}}{dp}=\frac{\Delta V}{\Delta H/T_{m}}$$

The volume change, Δ*V*, upon melting of the B-DNA construct in pure buffer solution can be calculated. We obtained Δ*V* ≈ +18 mL mol^−1^, which is about the molar volume of one water molecule, only. The positive sign of Δ*V* is in line with the observed stabilization of the folded duplex state upon pressurization. Such stabilization is quite expected as, in contrast to proteins, canonical DNA structures (having *T*_m_-values > 50 °C) are generally stabilized by hydrostatic pressure [18,19] owing to their dense packing and stabilization via H-bonding and π-π-stacking interactions, which are marginally affected by pressure, only. The volumetric properties of nucleic acids seem to be essentially determined by the state of hydration of the counterions that are accumulated in their vicinity, the specific contribution of the counterions depending on their identity (charge density, polarizability, size) and the structure of the nucleic acid sequence [18,19,23]. Significant stabilization was observed in the presence of high concentrations of the sodium and magnesium salts, which is even more pronounced at high pressure of 1500 bar, where the melting temperature reaches about ~97 °C in the presence of 250 mM NaCl. A similar observation was made in the presence of 250 mM MgCl_2_ for which no melting of the B-DNA was observed below 100 °C at 1500 bar. These data indicate a similar strong stabilization effect of the B-DNA by Na^+^ and Mg^2+^ with Cl^−^ as counterion at ambient pressure, a further increase in stability at high pressures for the Mg^2+^ cation, however. The comparison with the 250 mM Mg(ClO_4_)_2_ data indicate that the perchlorate anion, ClO_4_^−^, imposes a slight destabilizing effect compared to Cl^−^. The similarity of Clapeyron slopes, d*T*_m_/d*p*, suggests similar volume and enthalpy changes upon melting, however. The increase in Clapeyron slope in the presence of salts can be explained by an increase in Δ*V* and/or decrease in Δ*H*, which could be due to differential hydration effects in these salt solutions.

**Figure 2:**
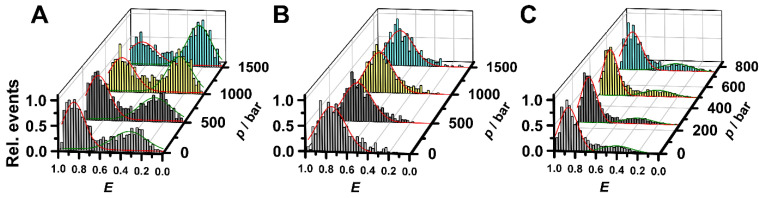
Pressure dependent FRET distribution histograms of the DNA-hairpin (**A**) in buffer, (**B**) in 6 mM MgCl_2_ and (**C**) in 6 mM Mg(ClO_4_)_2_. The buffer was 20 mM TrisHCl, pH 7.5. The measurements were carried out at 25 °C. Please note, that sm-FRET measurements in the high-pressure capillary are a bit noisier and broadened compared to the ambient pressure measurements on a coverslip (Figure 3).

To unveil the influence of typical Martian salts like perchlorates on the conformational dynamics of non-canonical nucleic acids at elevated pressure conditions, we studied their effect on the stability of a DNA-HP. DNA-hairpins are common secondary structure motifs that play important roles in gene expression, DNA recombination, and transposition [35]. To be able to differentiate between different conformational states of the system, we employed the single-molecule förster resonance energy transfer (sm-FRET) technique, which also allows pressure dependent measurements to be carried out using special pressure-resistant quartz capillaries. For experimental details, please refer to [28,29,30,31,32,33,34]. Peaks in the recorded FRET efficiency histograms are related to conformations with different spatial separations, *R*, of the two attached fluorescent dyes, yielding different FRET efficiencies, *E*, according to ${E=R_{0}^{6}\cdot (R_{0}^{6}+R^{6})}^{-1}$. The Förster radius, *R*_0_, is the distance at which 50% of the excited donor molecules will be deactivated; here, *R*_0_ = 6.5 nm for the fluorophores used, Atto 550 and Atto 647N. According to the FRET efficiency analysis, individual hairpins remain in a low (*E* ≈ 0.3) or a high (*E* ≈ 0.90) FRET state, which correspond to the open and the closed (native) conformation of the DNA-HP, respectively. We found that over the whole pressure range covered (1–1500 bar), the hairpin stays always in an equilibrium between the open and closed conformation in neat buffer solution. As seen in Figure 2A, upon pressurization, the structure of the DNA-HP gets destabilized, leading to a shift in the conformational equilibrium towards the open, unfolded state. At high hydrostatic pressure (1500 bar), the population of the open conformation increases up to ~60%, which corresponds to a volume change, Δ*V*, for the helix-to-coil transition of about −18 mL mol^−1^, in agreement with the literature data [32]. Significant changes were observed in the presence of MgCl_2_ and Mg(ClO_4_)_2_ salt. When adding even low salt concentrations such as 6 mM MgCl_2_, only the native, closed conformation is detectable in the FRET histogram at 1 bar, and the conformation remains stable even up to pressures of 1500 bar (Figure 2B). Conversely, in the case of 6 mM Mg(ClO_4_)_2_, both open and closed conformers are detectable at ambient pressure, the population of the closed and open states being about 80% and 20%, respectively (Figure 2C). Furthermore, unlike the scenario in neat buffer condition, the fraction of closed conformation of the DNA-HP remains essentially constant up to the maximum pressure reached, indicating that stabilization of the DNA-HP structure is not only driven by the divalent cation, but also affected by the corresponding counterion. Furthermore, the perchlorate leads to a small destabilizing effect of the native conformation of the DNA-HP compared to MgCl_2_, resulting in a small population of unfolded states. The population distribution does not change with pressure, suggesting that the magnitude of the volume change decreased, rendering Δ*V* negligible.

**Figure 3:**
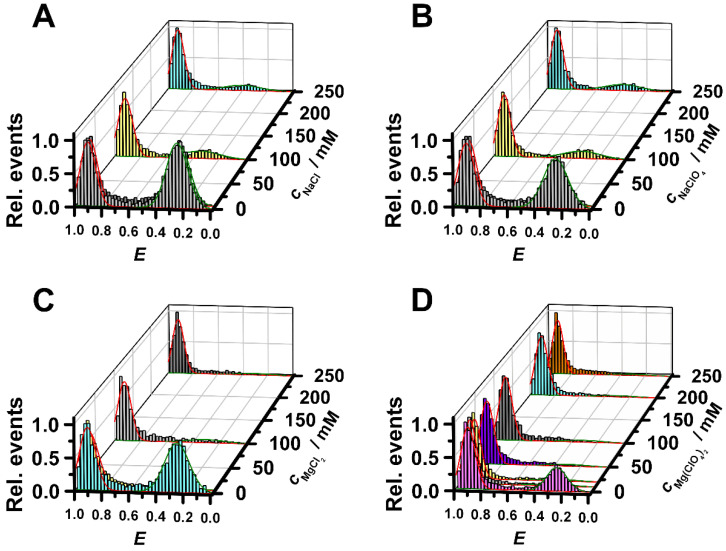
FRET histograms of the DNA-hairpin in the presence of different salt concentrations: (**A**) NaCl, (**B**) NaClO_4_, (**C**) MgCl_2_ and (**D**) 15 mM NaCl + Mg(ClO_4_)_2_ at ambient pressure and temperature (*T* = 25 °C).

Altogether, we found that the salt effects observed were dependent on the chemistry of the salt and the internal architecture of the nucleic acid structure. In the case of the canonical nucleic acid conformation, the double-stranded B-DNA, perchlorate anion lowers the stability of the double-strand compared to the chloride, probably due to weak (stacking) interactions of this highly polarizable, almost hydrophobic anion with nucleic acid bases. Furthermore, the stability in the presence of NaCl is higher compared to both MgCl_2_ and Mg(ClO_4_)_2_ at the same salt concentration. Hence, the stability is not only dependent on the cation but also on its counterion. On the other hand, the folded state of the DNA-HP is more efficiently stabilized in the presence of MgCl_2_ and Mg(ClO_4_)_2_ compared to the corresponding sodium salts, which suggests that the extra conformational stability is gained by the divalent cation, which is also in accordance with earlier studies [36] reporting that DNA and RNA helices are stabilized more effectively by Mg^2+^ than by Na^+^. The salt-dependent sm-FRET data presented for the DNA-HP in Figure 3 clearly show that the addition of NaCl and NaClO_4_ has a similar effect on the conformational stability of the DNA-HP, leaving a small population of unfolded states (~20%) still present at 250 mM salt concentration. Conversely, the MgCl_2_ and Mg(ClO_4_)_2_ salts lead to a more effective stabilization of the folded structure of the DNA-HP.

## 4. Concluding Remarks

Taken together, we found that significant stabilization of the B-DNA and DNA-HP is gained in the presence of all salts, even those including the chaotropic perchlorate anion, the degree of stabilization depending both on the cation and its counterion. The perchlorate imposes a destabilizing effect compared to the chloride salts, which is still small at high (250 mM) salt concentrations. Pressure has a minor influence on the conformational dynamics of the DNA-HP in the presence of high salt concentration. The stability of the B-DNA in the presence of high salt increases still significantly at high pressures, in particular in the presence of MgCl_2_. This suggests that the salt induces a more compact packing by reducing the repulsive interaction between the backbone’s phosphate groups through effective charge screening and different hydration characteristics of the DNA. Hence, these results show that Mars-relevant salts confer small-scale changes in the stability of nucleic acids at pressures up to the kbar level, which may imply some biochemical adaptations to adjust, but that nucleic acids remain fundamentally stable under potential subsurface Martian conditions. This suggests that biochemical adaptation to high pressure, perchlorate rich environments should predominantly be seen in lipid membranes and proteins as they exhibit higher sensitivity to such conditions than nucleic acids.

## Data Availability

The data presented in this study are available on request from the corresponding author.
